# A Novel Assay Allowing Drug Self-Administration, Extinction, and Reinstatement Testing in Head-Restrained Mice

**DOI:** 10.3389/fnbeh.2021.744715

**Published:** 2021-10-29

**Authors:** Kelsey M. Vollmer, Elizabeth M. Doncheck, Roger I. Grant, Kion T. Winston, Elizaveta V. Romanova, Christopher W. Bowen, Preston N. Siegler, Lisa M. Green, Ana-Clara Bobadilla, Ivan Trujillo-Pisanty, Peter W. Kalivas, James M. Otis

**Affiliations:** ^1^Department of Neuroscience, Medical University of South Carolina, Charleston, SC, United States; ^2^School of Pharmacy, University of Wyoming, Laramie, WY, United States; ^3^Langara College, Vancouver, BC, Canada; ^4^Hollings Cancer Center, Medical University of South Carolina, Charleston, SC, United States

**Keywords:** two-photon (2P), calcium imaging, addiction, ensembles, longitudinal tracking of individual cells

## Abstract

Multiphoton microscopy is one of several new technologies providing unprecedented insight into the activity dynamics and function of neural circuits. Unfortunately, some of these technologies require experimentation in head-restrained animals, limiting the behavioral repertoire that can be integrated and studied. This issue is especially evident in drug addiction research, as no laboratories have coupled multiphoton microscopy with simultaneous intravenous drug self-administration, a behavioral paradigm that has predictive validity for treatment outcomes and abuse liability. Here, we describe a new experimental assay wherein head-restrained mice will press an active lever, but not inactive lever, for intravenous delivery of heroin or cocaine. Similar to freely moving animals, we find that lever pressing is suppressed through daily extinction training and subsequently reinstated through the presentation of relapse-provoking triggers (drug-associative cues, the drug itself, and stressors). Finally, we show that head-restrained mice will show similar patterns of behavior for oral delivery of a sucrose reward, a common control used for drug self-administration experiments. Overall, these data demonstrate the feasibility of combining drug self-administration experiments with technologies that require head-restraint, such as multiphoton imaging. The assay described could be replicated by interested labs with readily available materials to aid in identifying the neural underpinnings of substance use disorder.

## Introduction

Multiphoton microscopy is a powerful tool used to help dissect the neurobiological substrates that coordinate behavior. By harnessing fluorescent imaging strategies based on genetically encoded constructs, its never-before-seen spatiotemporal resolution in living tissues enables observation and tracking of single neurons (e.g., calcium ion concentration or voltage changes; Tian et al., 2009; Chen et al., 2013; Lin and Schnitzer, 2016; Villette et al., 2019), neurotransmitter release (e.g., receptor-activation-based neurotransmitter sensors; Sun et al., 2018; Feng et al., 2019) and cell morphology (e.g., dendritic spine plasticity; Muñoz-Cuevas et al., 2013; Moda-Sava et al., 2019) in awake, behaving animals. Such technology could be transformative for studying substance use disorder (SUD), which is rooted in plasticity in the neural circuits that govern motivated behavior (Russo and Nestler, 2013). Unfortunately, experiments involving multiphoton imaging generally require head immobilization, which has prevented its integration with preclinical models of SUD.

Perhaps the most powerful preclinical model for SUD involves training animals to voluntarily self-administer drugs of abuse, an approach that has both predictive and construct validity for treatment outcomes and abuse liability (Shalev et al., 2002; Epstein et al., 2006; Haney and Spealman, 2008). In drug self-administration, animals are reinforced for performing an operant task (e.g., lever pressing) by drug delivery (e.g., intravenous), after which seeking behavior is extinguished through reward omission. Animals can then be tested for reinstatement of drug seeking in response to stimuli known to produce craving and relapse in humans, such as drug-associated cues, a single dose of the drug, or stressors. Distinct phases of self-administration can be used to study the cycle of intoxication, withdrawal, and drug seeking, which characterizes SUD (Koob and Le Moal, 1997) and is paralleled by neural plasticity in the brain’s reward circuits (Gardner, 2000). By pairing self-administration with bench approaches, critical factors underlying drug-seeking behavior have been identified at the cellular, molecular, and circuit levels (Koob and Le Moal, 1997; Kalivas and Volkow, 2005; Deadwyler, 2010; Russo et al., 2010). Despite these advances, our ability to precisely observe, longitudinally track, and manipulate these implicated factors in live, behaving animals has been limited.

Here we design and develop a new approach for drug self-administration in head-restrained mice, an assay that would allow simultaneous multiphoton imaging. Overall, we find that head-restrained mice will reliably learn to press an active, but not inactive, lever that results in the delivery of a tone cue followed by intravenous heroin or cocaine, but not saline. We find that omission of the cue and drug results in extinction learning, wherein active lever pressing is suppressed across days. Following extinction training, presentation of the drug-associated cue, the drug itself, or stressors cause reinstatement of drug seeking, as is typically observed in freely moving studies. Finally, we show that mice will similarly self-administer a sucrose reward, delivered orally, which is a common control used in drug self-administration experiments. Interestingly, cue presentation, but not drug or stress exposure, results in reinstatement of sucrose seeking after extinction. These results demonstrate the feasibility of head-restrained drug self-administration and control sucrose self-administration experiments in mice, enabling the integration of novel technologies that require head immobilization (e.g., two-photon microscopy) to study the neuronal mechanisms of SUD.

## Materials and Methods

### Subjects

Male and female C57BL/6J mice (8 weeks old/20 g minimum at study onset; Jackson Labs) were group-housed pre-operatively and single-housed post-operatively under a reversed 12:12-hour light cycle (lights off at 8:00am) with access to standard chow and water *ad libitum*. Experiments were performed in the dark phase and in accordance with the NIH Guide for the Care and Use of Laboratory Animals with approval from the Institutional Animal Care and Use Committee at the Medical University of South Carolina.

### Surgeries

Mice were anesthetized with isoflurane (0.8–1.5% in oxygen; 1 L/min) and placed within a stereotactic frame (Kopf Instruments) for head ring implantation surgeries. Ophthalmic ointment (Akorn), topical anesthetic (2% Lidocaine; Akorn), analgesic (Ketorlac, 2 mg/kg, ip), and subcutaneous sterile saline (0.9% NaCl in water) treatments were given pre- and intra-operatively for health and pain management. A custom-made ring (stainless steel; 5 mm ID, 11 mm OD) was adhered to the skull using dental cement and skull screws. Head rings were scored on the base using a drill for improved adherence. Following surgeries, mice received antibiotics (Cefazolin, 200 mg/kg, sc).

Drug self-administration mice were allowed at least 7 days of recovery from head ring implantation before catheterization occurred. Once recovered, mice were anesthetized as described above and implanted with indwelling intravenous catheters for drug self-administration. Catheters (Access Technologies #071709H) were custom-designed for mice, with 4.5 cm of polyurethane tubing (0.012′′ ID; 0.025′′ OD, rounded tip) between the sub-pedestal/indwelling end of the back-mounted catheter port (Plastics One #8I313000BM01) and silicone vessel suture retention bead, from which 1.0 cm extended intravenously via the right external jugular vein toward the right atrium of the heart. Larger tubing (1.5 cm of 0.025′′ ID; 0.047′′ OD) encased the smaller attached to the pedestal, and components were adhered by ultraviolet curation [catheter construction was based on Doncheck et al. (2018)]. Catheters were implanted subcutaneously using a dorsal approach, with back mounts externalized through ≤ 5 mm midsagittal incisions posterior to the scapulae and tubing running from the sub-pedestal base over the right scapula into the <1 mm incision in the right external jugular vein. Silk sutures adhered the catheter tubing retention bead to the external jugular at the site of incision and negative pressure-induced blood backflow was assured prior to closing skin incisions with non-absorbable monofilament sutures. All animals received analgesic, ophthalmic, and antibiotic treatments as described above in addition to topical antibiotic ointment and lidocaine (2%) jelly application to incisions. Mice were allowed to recover for a minimum of 1 week before behavioral experiments. Following implantation, catheters were flushed daily with heparinized saline (60 units/mL, 0.02 mL) to maintain patency. Animals with non-patent catheters were to be excluded from the study, however, all animals remained patent throughout acquisition; catheter patency was no longer monitored once the animals entered extinction. When necessary, patency was determined by giving mice an i.v. infusion of brevital (2 mg/mL, 0.02 mL).

### Self-Administration Chambers

Self-administration chambers were custom designed and created using readily available and cost-effective components. Each chamber was a two-door cabinet (24′′ × 16′′ × 30′′; NewAge Products; #50002) equipped with (1) soundproofing, (2) infusion pump, (3) restraint tube, (4) laptop with MATLAB and Arduino software, (5) breadboard, (6) head-fixation station, (7) operant levers, (8) speaker, and (9) an Arduino (see Supplementary Figure 1).

1. Soundproofing: Acoustic foam (2.5′′ × 24′′ × 18′′ UL 94, Professional Acoustics; 12′′ × 12′′ × 1.5′′ egg crate acoustic foam tiles, OBCO) was glued to the inner chamber.
2. Infusion pump: Arduino-controlled infusion pumps (Med Associates #PHM-100VS-2) were used for drug delivery. For drug delivery, a 3 mL syringe filled with drug (cocaine, heroin, or saline) was connected to tubing (Tygon, 0.02′′ ID; 0.06′′ OD) which could be fitted over backmounted i.v. catheters. For sucrose delivery, a 3 mL syringe filled with sucrose (12.5%) was connected to tubing (Tygon, 0.05′′ ID; 0.09′′ OD) that attached to a lick spout positioned in front of the mouse.
3. Restraint tube: Partial body restraint was used to avoid self-injury. Rotary grinders were used to make slits in 50 mL conical tubes (Fisher; 01-812-55) to allow for catheter port externalization while mice were partially restrained within head-fixation stations. The open end of the conical faced the lever box and was positioned to allow free range of movement for the front limbs.
4. Laptop: A laptop (Lenovo Ideapad 330S; 81F5006GUS) interfaced with other electronics via Arduino. Arduino- (Arduino 1.8.12) and MATLAB- (MathWorks) software were used to control equipment and record behavioral events.
5. Breadboards: An aluminum breadboard (12′′ × 18′ × 1/2′′) was used as the base to allow for chamber components to be screwed in place (ThorLabs; MB1218). A smaller aluminum breadboard (4′′ × 6′′ × 1/2′′) was used to secure the head-fixation station into place and to provide a behavioral platform (ThorLabs; MB4).
6. Head-fixation station: Head-fixation stations were custom-made (Clemson University Engineering Department) and attached to the breadboard. Stainless steel inverted square-edged U-frames with slits in the central crossbar allowed for mouse insertion by head rings. A second crossbar clamped the head rings down to prevent head movement.
7. Operant levers: Two operant levers (Honeywell; 311SM703-T) were cemented into a hollowed-out 12-well plate (Fisher; FB012928) and wired for active/inactive response functionality through the Arduino board. The levers extended outward by 5 cm from this “lever box” and the ends aligned centrally 3.5 cm from the edge of the restraint conical. Forelimb extension was required for mice to reach the levers.
8. Speaker: Arduino-controlled piezo buzzers (Adafruit; #1739) were positioned above the head-fixation frames for the delivery of auditory, reward-conditioned cues. The cue was initiated immediately following an active lever press during a non-timeout period.
9. Arduino board: Arduino (Arduino Uno Rev 3; A000066) interfaced with two electronic breadboards (Debaser Electronics; DE400BB1-1) for control of self-administration equipment.

### Behavioral Procedure

Mice were given at least 7 days to recover from their previous surgery (head ring implantation or catheterization) before beginning behavior. Once recovered, mice underwent 3 days of habituation, during which they were head-restrained for 30 min in the operant chambers without access to the levers. During acquisition, the operant levers were placed in front of the mice. Mice self-administering heroin, cocaine, or saline received intravenous infusions via tubing connecting the syringe pump and their i.v. catheters. Mice self-administering sucrose received liquid droplets via a lick spout placed in front of their mouth. Pressing the active lever resulted in immediate cue presentation (8 kHz, 2 s), followed by a gap in time (trace interval, 1 s), and finally intravenous drug or saline infusion (2 s; 12.5 μl) or sucrose droplet (2 s; 12.5 μl). The trace interval is included for isolation of sensory cue- and reward-related neural activity patterns during multiphoton imaging, similar to previous appetitive learning experiments (Otis et al., 2017; Grant et al., 2021). Reinforced active lever presses also resulted in a timeout period wherein further active lever presses were recorded but not reinforced with the cue or reward. Inactive lever presses resulted in neither cue nor reward delivery.

#### Heroin Self-Administration

Mice underwent 14 days of heroin self-administration on a fixed ratio 1 (FR1) schedule of reinforcement using a decreasing dose design (Day 1–2: 0.1 mg/kg/12.5 μl heroin, 10 infusion maximum; Day 3–4: 0.05 mg/kg/12.5 μl heroin, 20 infusion maximum; Day 5–14: 0.025 mg/kg/12.5 μl heroin, 40 infusion maximum), for a maximum of 1 mg/kg of heroin per session (similar to previously described experiments in freely moving mice; Corre et al., 2018). Due to quick responding on the active lever, mice were capped to receiving 1 mg/kg per session to prevent overdose. Concurrently, a separate group of mice underwent 14 days of saline (12.5 μl infusions; 40 infusion maximum) self-administration sessions on an FR1 schedule of reinforcement. Session durations for both heroin and saline self-administering mice were a maximum of 2 h. Following acquisition, heroin and saline self-administering mice underwent 2-h extinction training sessions, wherein active lever presses resulted in neither cue nor drug delivery until extinction criteria were reached. Extinction criteria were determined *a priori*, defined as (1) ≥ 10 days of extinction training and (2) 2 of the last 3 days resulting in ≤20% of the average active lever pressing observed during the last 2 days of acquisition. Heroin self-administering mice that did not reach extinction criteria were excluded from analyses and subsequent reinstatement testing (*n* = 4/28). Saline self-administering mice were immediately tested after the 10th day of extinction due to low responding during acquisition, and thus an inability to “extinguish” lever pressing. Due to initial piloting, only a subset of saline self-administering mice underwent extinction and reinstatement testing (*n* = 4). Following establishment of extinction criteria, heroin and saline self-administering mice underwent reinstatement testing in a pseudorandom order, for cue-, drug-, yohimbine-, predator odor-, or saline-induced reinstatement tests. Animals were tested first for cue-induced reinstatement, but the subsequent stimuli were tested using a counterbalanced design. For cue-induced reinstatement testing, active lever presses resulted in cue presentation in a manner identical to acquisition, but drug infusions were excluded. For drug-primed reinstatement, heroin (1 mg/kg, i.p.) was delivered immediately prior to the reinstatement session. A heroin-primed reinstatement dose-response pilot study revealed this dose produced responding most comparable to that observed during both acquisition and other reinstatement tests (data not shown). For reinstatement testing in response to a pharmacological stressor, yohimbine (0.625 mg/kg, i.p.; Sigma Chemical; King and Becker, 2019) was given 30 min before the session. Yohimbine, the alpha-2-noradrenergic receptor antagonist, may influence behavior in a manner that is not purely stress-related (e.g., Chen et al., 2015). Thus, we included an additional stress-induced reinstatement test provoked by the predator odor 2,3,5-trimethyl-3-thiazoline (TMT), a synthetically derived component of fox feces chosen due to its ethological relevance and validity as a stressor (Janitzky et al., 2015). For predator odor-induced reinstatement, TMT (30 μL; 1% v/v ddH2O) was placed in front of each head-restrained mouse on a gauze pad inside a container connected to a vacuum (to restrict odor spread) for 15 min and removed immediately before behavioral testing (King and Becker, 2019). For saline-primed reinstatement, 0.9% NaCl (10 mL/kg, i.p.) was delivered immediately before behavioral testing. Heroin and saline self-administering mice were re-extinguished to criteria between reinstatement tests. Due to initial piloting, not all heroin self-administering mice that completed acquisition and extinction underwent all 5 reinstatement tests.

#### Cocaine Self-Administration

Mice underwent 14 days of 2-hour cocaine (0.75 mg/kg/12.5 μl cocaine infusion; 40 infusion maximum) self-administration sessions on an FR1 schedule of reinforcement similar to that described in freely moving mice (Heinsbroek et al., 2017). The criteria for extinction were defined in the same manner as for heroin (see above), and no mice were excluded for failure to reach criteria. Mice underwent reinstatement testing using a counterbalanced design similar to the methods described above for heroin self-administration reinstatement testing, with the exception of drug-primed reinstatement involving cocaine (5 mg/kg, i.p.) rather than heroin injections immediately before the session. A subset of cocaine self-administering mice (*n* = 4/15) did not undergo extinction or reinstatement testing due to initial piloting. The saline self-administering mice detailed above (see section “Heroin Self-administration”) underwent acquisition concurrently with cocaine self-administration mice and were thus included as a control for both experiments.

#### Sucrose Self-administration

Mice underwent 14 days of 2-hour sucrose self-administration sessions on an FR1 schedule of reinforcement. As sucrose self-administration is often used as a control for drug self-administration, sucrose self-administration mice followed an intake design similar to the methods described above for heroin self-administration animals. While sucrose droplets were capped (Day 1–2: 12.5 μl of 12.5% sucrose, 10 droplets maximum; Day 3–4: 12.5 μl of 12.5% sucrose, 20 droplets maximum; Day 5–14: 12.5 μl of 12.5% sucrose, 40 droplets maximum), the volume of sucrose delivered per reward did not change. Following the last day of acquisition, sucrose self-administering mice underwent extinction and reinstatement testing using the same criteria and pseudorandom design, respectively, as described for heroin self-administration (see above). No mice were excluded for failure to reach extinction criteria.

### Data Collection and Statistics

Parameters for behavioral sessions were set using a custom MATLAB graphical user interface that controlled an Arduino and associated electronics. Data were recorded using MATLAB, extracted using Python, and analyzed and graphed using GraphPad PRISM (version 8), and illustrated using Adobe Illustrator. Analysis of variance (ANOVA; 2-way or 3-way repeated measurers) or *t*-tests (paired) were used to analyze data collected for the experiments. Independent variables included lever (active vs. inactive), day (e.g., extinction vs. reinstatement), and sex (male vs. female). Sidak’s *post hoc* tests were used following significant main effects and interactions within the ANOVA analyses. Due to initial electrical issues with the Arduino circuit board, data showed that some mice exhibited lever responding exceeding what is feasible (∼60 Hz). This electrical issue was due to improper grounding of the electrical components within the behavioral chamber, and the subsequent static resulted in unachievable lever press counts. We promptly corrected the drifting ground by ensuring the electrical connection between levers and the Arduino circuit board was secure. To remove the variance caused by this electrical issue, we excluded outliers greater than two standard-deviations away from the mean for each session (14 out of 1022 total acquisition sessions from all drug and sucrose self-administration animals). Additionally, during initial troubleshooting phases of the experiments we lost three files that were not saved to the computer, and thus that data is not included in acquisition (3 out of 392 heroin self-administration acquisition sessions).

## Results

### Head-Restrained Heroin Self-Administration

#### Acquisition

**FIGURE 1:**
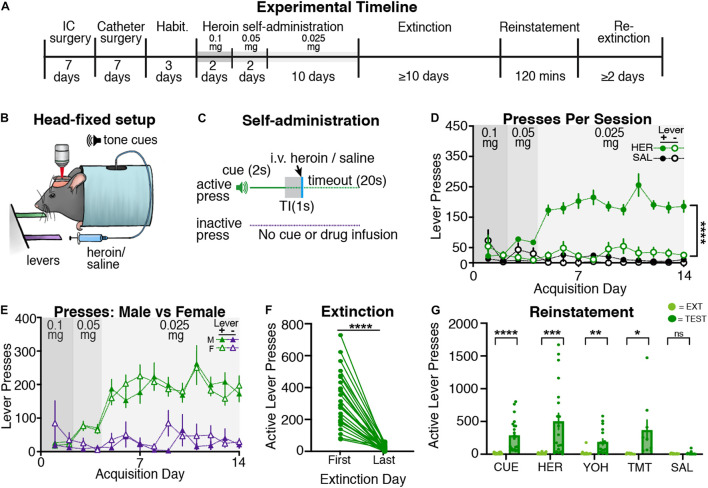
Head-restrained mice self-administer heroin and display extinction and reinstatement of heroin seeking. **(A)** An experimental timeline demonstrating the behavioral procedure used for head-restrained heroin self-administration. **(B)** Illustration of head fixation for heroin self-administration experiments. Active lever presses resulted in a tone cue that predicted intravenous heroin [at doses of 0.1 mg/kg (day 1–2), 0.05 mg/kg (day 3–4), or 0.025 mg/kg (day 5–14)] or intravenous saline. **(C)** Schematic for heroin/saline self-administration experiments, where active but not inactive lever presses resulted in a tone cue (2s), a gap in time (TI, trace interval; 1s), an infusion of heroin or saline, and a timeout period (20s). **(D)** Data showing that heroin self-administering mice pressed the active lever more than the inactive lever (****p < 0.001), whereas saline self-administering mice did not. **(E)** A comparison of active and inactive lever pressing across sexes. Male and female heroin self-administering mice showed no differences in lever pressing across acquisition. **(F)** During extinction, heroin self-administering mice significantly decreased active lever pressing from the first day of extinction to the last (****p < 0.001). **(G)** Data showing that mice displayed cue- (****p < 0.001), heroin- (***p = 0.001), yohimbine- (**p = 0.002), and predator odor (TMT; *p = 0.048)-induced reinstatement of active lever pressing as compared with the previous extinction test. Mice did not reinstate following an injection of saline.

Following recovery from surgery and habituation (see Figure 1A for timeline), mice began head-restrained heroin (*n* = 28; 13 males and 15 females) or saline self-administration (*n* = 8; 5 males and 3 females; Figures 1B,C). Heroin self-administering mice learned to reliably press the active lever more than the inactive lever across acquisition, but saline animals did not exhibit a preference for pressing either lever (Figure 1D). A three-way ANOVA revealed a significant effect of drug [F(1,68) = 62.0, *p* < 0.001] and a drug by lever interaction [F(1,68) = 35.5, *p* < 0.001], but no drug by lever by day interaction [F(13,862) = 1.67, *p* = 0.06]. Following two-way ANOVAs (day × lever separated for each group) revealed an effect of lever for heroin self-administering mice [F(1,54) = 133.5, *p* < 0.001], but not for saline self-administering mice [F(1,14) = 0.01, *p* = 0.92]. The heroin self-administering mice also received more infusions as compared with the saline self-administering mice (Supplementary Figure 2A), as a two-way ANOVA revealed an effect of drug [F(1,34) = 2655; *p* < 0.001] and day by drug interaction [F(13,434) = 147.7, *p* < 0.001]. *Post hoc* tests revealed that heroin self-administering mice began receiving significantly more infusions starting on day 2 of acquisition (day 1, *p* = 0.68; days 2–14, *ps* < 0.05). Thus, mice rapidly and readily acquired heroin self-administration while head restrained.

#### Extinction and Reinstatement

Following acquisition, heroin self-administering mice underwent extinction training, wherein active lever presses no longer resulted in heroin or cue delivery. We find that head-restrained, heroin self-administering mice significantly decrease active lever pressing [Figure 1F; t(27) = 8.95; *p* < 0.001], but not inactive lever pressing [Supplementary Figure 3A; t(27) = 0.92; *p* = 0.37] from the first to last day of extinction. Next, heroin self-administering mice underwent cue- (*n* = 22; male = 10, female = 12), heroin- (*n* = 20; male = 8, female = 12), yohimbine- (*n* = 17; male = 8, female = 9), predator odor- (*n* = 9; male = 4, female = 5), and/or saline- (*n* = 14; male = 7, female = 7) induced reinstatement tests (Figure 1G). Paired *t*-tests revealed that heroin self-administering mice significantly increased active lever pressing during cue- [t(21) = 5.41; *p* < 0.001], heroin- [t(19) = 3.97; *p* = 0.001], yohimbine- [t(16) = 3.87; *p* = 0.002], and predator odor-induced [t(8) = 2.33; *p* = 0.048] reinstatement tests, but not following an injection of saline [t(13) = 0.89; *p* = 0.39], relative to responding during the previous extinction session. Heroin self-administering mice did not increase inactive lever pressing during cue- [t(21) = 1.61; *p* = 0.12], heroin- [t(19) = 0.77; *p* = 0.45], yohimbine- [t(16) = 1.48; *p* = 0.15], predator odor- [t(8) = 0.69; *p* = 0.50], or saline- [t(13) = 1.58; *p* = 0.13] induced reinstatement tests relative to the previous extinction session (Supplementary Figure 3B). Overall, we find that head-restrained animals readily self-administer heroin, extinguish lever pressing, and display reinstatement of heroin seeking similar to freely moving mice (Corre et al., 2018).

A subset of saline self-administering mice (*n* = 4) underwent extinction training and reinstatement testing despite displaying low levels of lever responding throughout acquisition. As a result of this low responding, saline self-administering mice did not show a significant decrease of active lever pressing [t(3) = 0.0; *p* > 0.99] from the first day of extinction to the last (Supplementary Figure 4A). Additionally, saline self-administering mice did not increase active lever pressing to any testing conditions [Supplementary Figure 4B; *t*-tests, cue: t(3) = 0.86, *p* = 0.45; heroin: t(3) = 1.89, *p* = 0.15; yohimbine: t(3) = 2.29, *p* = 0.11; saline: t(3) = 1.13, *p* = 0.34]. These data are consistent with previous findings showing that saline self-administering rodents will not extinguish or reinstate active lever pressing (Kruyer et al., 2019; Chioma et al., 2021).

#### Sex Differences

During acquisition, we did not observe sex differences in lever press rates for heroin self-administering mice (Figure 1E), as a three-way ANOVA revealed no effect of sex, sex by lever interaction, or sex by lever by day interaction (*F*-values < 0.83; *p*s > 0.50). Male and female heroin self-administering mice also received a similar number of infusions (Supplementary Figure 2A) throughout acquisition, as confirmed by a two-way ANOVA revealing no effect of sex or sex by drug interaction (*F*-values < 1.0, *p*s > 0.5). During extinction, we find male and female mice display equivalent active lever presses from the first to last day (Supplementary Figure 2B; two-way ANOVA, effects of sex: *F*-values < 0.3, *p*s > 0.5). Lastly, we did not observe sex differences in reinstatement responding for heroin self-administering mice (Supplementary Figure 2C; two-way ANOVAs, effects of sex: *F*-values < 2.4, *p*s > 0.1; sex by day interactions: F-values < 1.45, *p*s > 0.25).

### Head-Restrained Cocaine Self-Administration

#### Acquisition

**FIGURE 2:**
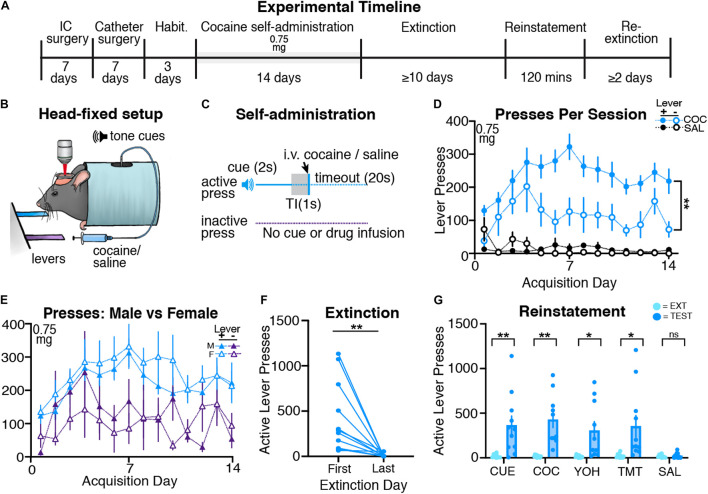
Head-restrained mice self-administer cocaine and display extinction and reinstatement of cocaine seeking. **(A)** An experimental timeline demonstrating the behavioral procedure used for head-restrained cocaine self-administration. **(B)** Illustration of head fixation for cocaine self-administration experiments. Active lever presses resulted in a tone cue that predicted intravenous cocaine (0.75 mg/kg on all days) or saline. **(C)** Schematic for cocaine self-administration experiments, where active but not inactive lever presses resulted in a tone cue (2s), a gap in time (TI, trace interval; 1s), an infusion of cocaine or saline, and a timeout period (20s). **(D)** Data showing that cocaine self-administering mice pressed the active lever more than the inactive lever (**p = 0.002). **(E)** A comparison of active and inactive lever pressing across sexes. Male and female cocaine self-administering mice showed no differences in lever pressing across acquisition. **(F)** All cocaine self-administering mice significantly decreased active lever pressing from the first day of extinction to the last (**p = 0.006). **(G)** Mice displayed cue- (**p = 0.007), cocaine- (**p = 0.002), yohimbine- (*p = 0.017), and predator odor (TMT; *p = 0.019)- induced reinstatement of active lever pressing as compared with the previous extinction test. Mice did not reinstate following an injection of saline.

Following surgery and habituation (see Figure 2A for timeline), mice (*n* = 15; 8 males and 7 females) underwent head-restrained cocaine self-administration (Figures 2B,C). Cocaine self-administering mice learned to press the active lever more than the inactive lever across acquisition, and when compared to saline self-administering animals, showed significantly more active lever pressing (Figure 2D). A three-way ANOVA revealed a significant effect of drug [F(1,42) = 48.51, *p* < 0.001] and a drug by lever interaction [F(1,42) = 6.15, *p* < 0.02], but no drug by lever by day interaction [F(13,512) = 0.19, *p* = 0.99]. Following two-way ANOVAs (day × lever separated for each group) revealed an effect of lever for cocaine self-administering mice [F(1,28) = 11.91, *p* = 0.002], but not for saline self-administering mice [F(1,14) = 0.01, *p* = 0.92]. Cocaine self-administering mice also received significantly more infusions than saline self-administering mice throughout acquisition (Supplementary Figure 2D), with a mixed-model two-way ANOVA showing an effect of drug [F(1,21) = 1564, *p* < 0.001], but no interaction between day and drug [F(13,266) = 0.70, *p* = 0.67]. Thus, mice reliably acquire cocaine self-administration behavior while head restrained.

#### Extinction and Reinstatement

Following cocaine self-administration, mice underwent extinction until criteria were met (*n* = 11; 6 males and 5 females). A paired *t*-test confirmed that cocaine self-administering mice significantly decreased both active [t(10) = 3.48; *p* = 0.006; Figure 2F] and inactive [t(10) = 2.58; *p* = 0.027; Supplementary Figure 3C] lever pressing from the first day of extinction to the last day. Next, mice underwent cue- (*n* = 11; 6 males, 5 females), cocaine- (*n* = 10; 6 males, 4 females), yohimbine- (*n* = 10; 6 males, 4 females), predator odor- (*n* = 11; 6 males, 5 females), and saline- (*n* = 10; 6 males, 4 females) induced reinstatement tests (Figure 2G). Paired *t*-tests revealed that, compared to the respective prior day’s extinction responding, mice significantly increased active lever pressing during cue- [t(10) = 3.34; *p* = 0.007], cocaine- [t(9) = 4.38; *p* = 0.002], yohimbine- [t(9) = 2.93; *p* = 0.017], and predator odor-induced [t(10) = 2.79; *p* = 0.019] reinstatement tests, but not but not following an injection of saline [t(9) = 0.77; *p* = 0.45]. Cocaine mice did not increase inactive lever pressing during cue- [t(10) = 1.88; *p* = 0.08], cocaine- [t(9) = 0.98; *p* = 0.34], yohimbine- [t(9) = 1.09; *p* = 0.29], predator odor- [t(10) = 1.85; *p* = 0.08], or saline-induced [t(9) = 0.31; *p* = 0.76] reinstatement tests relative to inactive lever responding during the most recent extinction session [Supplementary Figure 3D]. Overall, we find that head-restrained animals self-administer cocaine, extinguish lever pressing, and display reinstatement of cocaine seeking similar to freely moving mice (Heinsbroek et al., 2017).

#### Sex Differences

During acquisition, we did not observe sex differences in lever press rates for cocaine self-administering mice (Figure 2E), as a three-way ANOVA revealed no effect of sex, sex by lever interaction, or sex by lever by day interaction (*F*-values < 1.17; *p*s > 0.3). Male and female cocaine self-administering mice also received a similar number of infusions (Supplementary Figure 2D) during acquisition, as confirmed by a two-way ANOVA showing no effect of sex or sex by drug interaction (*F*-values < 1.3, *p*s > 0.6). During extinction, males and females display a similar number of active lever presses from the first to last day (Supplementary Figure 2E; two-way ANOVA, effects of sex: *F*-values < 0.1, *p*s > 0.5). We did not observe sex differences during the cue-, cocaine-, yohimbine-, and predator odor-induced reinstatement tests (two-way ANOVAs: main effect and interactions including sex: *F*-values < 0.82, *p*s > 0.05), but did see a significant effect of sex for the control injection of saline [F(1,8) = 7.92, *p* = 0.023; Supplementary Figure 2F]. *Post hoc* tests revealed that males pressed the active lever significantly more than females on the test day (*p* = 0.014), but not the previous extinction day (*p* = 0.974).

### Head-Restrained Sucrose Self-Administration

#### Acquisition

**FIGURE 3:**
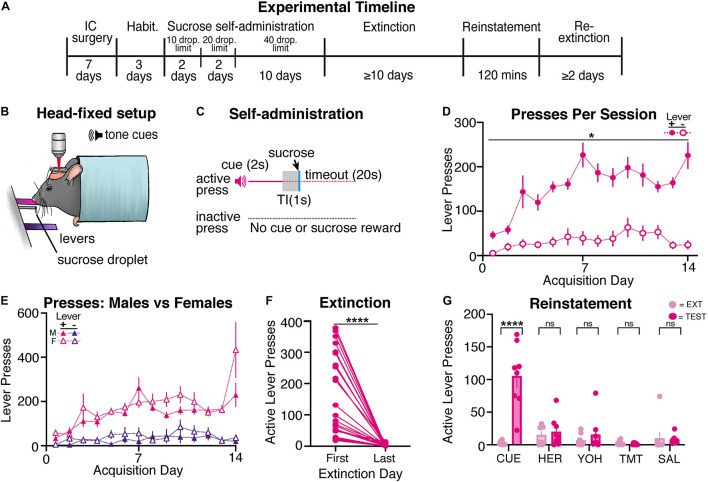
Head-restrained mice self-administer sucrose and display reinstatement of sucrose seeking to the reward-associated cue. **(A)** An experimental timeline demonstrating the behavioral procedure used for head-restrained sucrose self-administration. **(B)** Illustration of head fixation for sucrose self-administration experiments. Active lever presses resulted in a tone cue that predicted a sucrose droplet (12.5 μl of 12.5% sucrose). **(C)** Schematic for sucrose self-administration experiments, where active but not inactive lever presses resulted in a tone cue (2s), a gap in time (TI, trace interval; 1s), a palatable sucrose droplet, and a timeout period (20s). **(D)** Sucrose self-administering mice pressed the active lever significantly more than the inactive lever on each day of acquisition (*ps < 0.05). **(E)** A comparison of active and inactive lever pressing across sexes. Male and female sucrose self-administering mice showed no differences in lever pressing across acquisition. **(F)** All sucrose self-administering mice significantly decreased active lever pressing from the first day of extinction to the last (****p < 0.001). **(G)** Sucrose self-administering mice displayed an increase in active lever pressing to cue- (****p < 0.001), but not heroin-, yohimbine-, predator odor (TMT)-, or saline-induced reinstatement tests.

Since drug self-administration protocols often include natural reward (sucrose) self-administration as a control, we determined whether mice would self-administer liquid sucrose droplets within our head-restrained model. Following recovery from surgery and habituation (see Figure 3A for timeline), mice (*n* = 32; 15 males, 17 females) began head-restrained sucrose self-administration (Figures 3B,C), using a similar behavioral protocol as the previous heroin self-administration experiment. Sucrose self-administering mice learned to reliably press the active lever more than the inactive lever across acquisition (Figure 3D). A mixed-model two-way ANOVA showed a day by lever interaction [F(13,800) = 3.93, *p* < 0.001], and *post hoc* tests revealed that mice pressed the active lever more than the inactive lever during each day of acquisition (days 1–14, *ps* < 0.05). Sucrose self-administering mice often reached the maximum number of sucrose droplets across all sessions (Supplementary Figure 2G). Thus, sucrose self-administering mice were able to reliably acquire lever pressing for sucrose rewards while being head-restrained.

#### Extinction and Reinstatement

Following sucrose self-administration, mice underwent at least 10 days of extinction until criteria were met (*n* = 26, 14 males, 12 females). Sucrose self-administering mice significantly decreased active lever pressing [Figure 3F; t(25) = 5.63, *p* < 0.001], but not inactive lever pressing [Supplementary Figure 3E; t(25) = 1.50, *p* = 0.15], from the first day of extinction to the last. Following extinction, sucrose self-administering mice underwent cue- (*n* = 8; 4 males, 4 females), heroin- (*n* = 8; 3 males, 5 females), yohimbine- (*n* = 8; 3 males, 5 females), predator odor- (*n* = 8; 4 males, 4 females), and saline- (*n* = 8; 4 males, 4 females) induced reinstatement tests (Figure 3G). Paired t-tests revealed that, compared to the previous extinction session, mice significantly increased active lever pressing during cue-induced reinstatement [t(7) = 5.93, *p* < 0.001], but not during heroin- [t(7) = 0.55, *p* = 0.60], yohimbine- [t(7) = 0.76, *p* = 0.48], predator odor- [t(7) = 1.18, *p* = 0.28], or saline-induced [t(7) = 0.21, *p* = 0.84] reinstatement tests. Sucrose mice did not increase their inactive lever pressing during any reinstatement tests (Supplementary Figure 3F; *t*-values < 1.5, *p*s > 0.15). Overall, we find that head-restrained animals will self-administer sucrose, extinguish lever pressing, and display reinstatement of sucrose seeking to the sucrose-associated cue similar to freely moving mice (Bobadilla et al., 2020).

#### Sex Differences

Male and female mice did not differ in lever pressing during acquisition (Figure 3E), as a three-way ANOVA showed that, while there was an effect of sex [F(1,60) = 4.29, *p* = 0.04], there was no sex and lever interaction [F(1,60) = 0.12, *p* = 0.73] or day by sex by lever interaction [F(13,774) = 1.11, *p* = 0.34]. Furthermore, *post hoc* tests revealed that there were no sex differences for active (days 1–14, *p*s > 0.66) or inactive (days 1–14, *p*s > 0.35) lever pressing across acquisition. We find that male and female mice also receive a similar number of sucrose droplets (Supplementary Figure 2G) throughout acquisition (two-way ANOVA, effects of sex: *F*-values < 0.3, *p*s > 0.6). During extinction, males and females displayed a similar number of active presses on each day (Supplementary Figure 2H; effects of sex: *F*-values < 2.3, *p*s > 0.12). Additionally, we did not observe sex differences during any reinstatement tests (Supplementary Figure 2I; effects of sex: *F*-values < 2.1, *p*s > 0.12).

## Discussion

Here we demonstrate that mice will readily self-administer heroin and cocaine, but not saline, while head restrained through active lever pressing behavior. Mice will extinguish active lever pressing when the drug and drug-associated cues are omitted. After extinction, mice with drug experience will resume drug seeking upon re-exposure to stimuli known to provoke relapse in humans (i.e., drug-associated cues, the drug itself, and stressors; Kalivas and Volkow, 2005). However, we find that sucrose self-administering mice will only resume seeking following presentation of the reward-associated cue, whereas heroin and stressors do not provoke sucrose seeking. These findings indicate that our head-restrained procedure may share similar construct and predictive validity as drug self-administration assays in freely moving rodents (Epstein et al., 2006; Sanchis-Segura and Spanagel, 2006). Furthermore, this study demonstrates the feasibility of combining preclinical drug self-administration experiments with novel technologies that require head immobilization.

### Head-Restrained vs. Freely Moving Drug Self-Administration

Similar to freely moving drug self-administration studies, our head-restrained drug self-administration procedure allows mice to acquire, extinguish, and reinstate drug seeking behaviors. Despite these similarities, we find that head-restrained mice display some differences that are important to consider. Most notably, the number of lever presses is generally higher when comparing our results with experiments in freely moving mice (Heinsbroek et al., 2017). This increase is likely a result of immobilization, which isolates mice in front of the levers. Furthermore, the stress involved in head restraint could be a factor, as stress is known to facilitate and provoke drug seeking and taking (Piazza and Le Moal, 1998). An additional difference that we observe is a relatively high inactive lever press rate during acquisition for cocaine self-administering mice. This could be due to the behavioral sensitization caused by repeated cocaine exposure, an effect that substantially increases movement in mice (for review, see Steketee and Kalivas, 2011). It is possible that head restraint limits increased motor activity primarily to the forelimbs, resulting in stereotyped inactive lever pressing. Despite the increase in inactive pressing, it is important to note that head-restrained cocaine self-administering mice discriminate between active and inactive levers, suggesting goal-directed behavioral responding.

### Sex Differences During Head-Restrained Drug Self-Administration

Sex differences are often described in disorders associated with dysfunctional reward seeking (Fattore et al., 2008; Galmiche et al., 2019), with females displaying elevated cravings, relapse susceptibility, and loss of control over intake as compared with male counterparts (Rubonis et al., 1994; Robbins et al., 1999; Kippin et al., 2005; Striegel-Moore et al., 2009; Moran-Santa Maria et al., 2014). Sex differences have accordingly been found in rodent drug and sucrose self-administration experiments, wherein females reportedly show an enhanced rate of acquisition of drug taking (Lynch et al., 2001; Roth et al., 2002; Hu et al., 2004; Jackson et al., 2006), and greater reinstatement of drug (Fuchs et al., 2005; Anker and Carroll, 2010; Feltenstein et al., 2011) and sucrose seeking (Wei et al., 2021). However, a lack of sex differences in these behaviors have also been reported (Roth and Carroll, 2004; Doncheck et al., 2020). Within our head-restrained model, we find that males and females acquire cocaine, heroin, and sucrose self-administration at equivalent rates and display comparable levels of reinstatement. While head restraint limits the behavioral repertoire exhibited in freely moving animals, which may influence complex factors to differentially promote seeking in both sexes, our head-restrained procedure could be adapted for sex-specific studies. For example, allowing head-restrained mice to have extended drug access could be advantageous for revealing sex differences, as others have shown that females will significantly elevate drug intake during long-access cocaine or heroin self-administration (Roth and Carroll, 2004; Towers et al., 2019). To study propensity to relapse, our procedure could be modified to include abstinence, as it has been reported that females will exhibit greater sucrose (Wei et al., 2021) and drug-seeking reinstatement following a period of forced abstinence (Anker and Carroll, 2010; Feltenstein et al., 2011). Regardless of behavioral sex differences, when coupled with *in vivo* imaging techniques, our head-restrained procedure could provide insight into the sex-specific neurophysiology that underlies acquisition and reinstatement to drug seeking.

### Applications of Head-Restrained Drug Self-Administration

The field of preclinical *in vivo* neuroimaging has seen rapid innovation within recent years, facilitated by the development and improvement of imaging technologies and *in vivo* sensors. For example, through the application of head-restraint, scientists have been able to perform functional MRI (Chang et al., 2016; Stenroos et al., 2018; Desjardins et al., 2019; Liu et al., 2020), functional ultrasound (Brunner et al., 2021; Wang et al., 2021), wide-field (Kim et al., 2016; Silasi et al., 2016; Yoshida et al., 2018) and multiphoton fluorescence (Otis et al., 2017, 2019; Namboodiri et al., 2019; Rossi et al., 2019; Grant et al., 2021) imaging experiments in awake rodents. Coupling innovative preclinical imaging techniques with behavioral tasks can provide an extraordinary view into how complex neural systems mediate relevant behavioral states. Here, we combine head-restraint with an operant drug self-administration task so that we may investigate the precise neuroadaptations associated with the development of SUD. Pairing operant drug self-administration with the described *in vivo* imaging techniques could aid in elucidation of the neuronal underpinnings of SUD.

### Using Multiphoton Imaging to Track Activity and Morphological Plasticity in Cell-Type Specific Neurons From the Onset of Drug Use to Relapse

The development of SUD involves complex and long-lasting changes to activity (Koob and Volkow, 2016) and structural plasticity (for review, see; Russo et al., 2010; Spiga et al., 2014; Wolf, 2016; Kruyer et al., 2020) in brain reward circuitry, but how these adaptations develop in cell-type specific neurons and predict relapse vulnerability is unknown. With the advent of multiphoton microscopy (Denk et al., 1990) along with genetically encoded calcium indicators (Chen et al., 2013), we can now longitudinally measure activity (Otis et al., 2017, 2019; Namboodiri et al., 2019; Rossi et al., 2019; Grant et al., 2021) and visualize cell morphology (Muñoz-Cuevas et al., 2013; Moda-Sava et al., 2019) in deep brain, cell-type specific neurons for weeks to months in awake, behaving animals. This combinatorial approach can be exploited to monitor activity not only in hundreds to millions of neuronal cell bodies simultaneously (Dombeck et al., 2007; Kim et al., 2016), but also in dendrites (Lavzin et al., 2012), dendritic spines (Chen et al., 2011), and axons (Lovett-Barron et al., 2014; Otis et al., 2019). Although calcium indicators are by far the most commonly used for visualizing activity, other sensors are available and under continued development for visualization of ground-truth voltage (Gong et al., 2015), neurotransmitter release and binding (Nguyen et al., 2010; Jing et al., 2018; Marvin et al., 2018, 2019); molecular signaling (Greenwald et al., 2018; Muntean et al., 2018), and more (Marvin et al., 2011; Aper et al., 2016; Díaz-García et al., 2019). These powerful technologies could be combined with drug self-administration studies in head-restrained mice to provide an unparalleled view into the abnormal neural circuit activity patterns and the morphological adaptations that arise from the onset of drug use to relapse.

### Evaluating the Function of Neuronal Ensemble Activity Patterns and Morphological Plasticity in Drug Use and Seeking

Studies using multiunit recordings and calcium imaging technologies often reveal complex activity patterns within single brain regions during natural reward (Otis et al., 2017, 2019; Grant et al., 2021) and drug reward-seeking (Drouin and Waterhouse, 2004; Ahmad et al., 2017; Siciliano et al., 2019). These heterogeneous activity patterns are not just common at the population level, but also at the level of neuronal subpopulations, as recordings from genetically defined or projection-specific neurons also reveal profound cell-to-cell variability (Seong and Carter, 2012; Dembrow and Johnston, 2014; Al-Hasani et al., 2015; Yang et al., 2018). The inherent complexity of neuronal circuit activity patterns has made it difficult to selectively manipulate unique neuronal ensembles to determine their function for behavior. However, existing and emerging technologies that combine multiphoton microscopy with optogenetics now allow for manipulation of activity in experimenter-defined neurons in 3-dimensional space (Yang et al., 2018; Marshel et al., 2019). Furthermore, multiphoton laser-directed lesion experiments have been employed at the level of cell bodies, axons, dendrites, and dendritic spines *in vivo* (Allegra Mascaro et al., 2010; Canty et al., 2013; Hill et al., 2017; Park et al., 2019). These technologies could now be combined with drug self-administration studies to identify the function of unique neuronal ensembles and morphological plasticity for drug use and seeking.

### Future Directions and Limitations

A potential limitation of our protocol is that it is unknown whether head restraint produces greater basal stress levels compared to freely moving, albeit catheter-tethered mice. This caveat is important given that chronic stress can lead to escalation of drug intake (Mantsch and Katz, 2007), which may produce behavioral phenotypes akin to those observed in animals with a greater history of intake (i.e., “long-access” or “extended-access,” rather than “short-access,” animals; Mantsch et al., 2016). Future modifications to our approach could be beneficial for reducing the possible effects of stress on drug seeking and allow for improved behavioral resolution within the task itself. For example, inclusion of a running wheel, treadmill, or trackball would provide an avenue for mice to move while head restrained and would also provide a behavioral readout of locomotor activity – a behavioral variable that is often studied due to its robust modulation by repeated drug use and correlation with addiction vulnerability (Piazza et al., 1989; Zhou et al., 2019). Notably, as our mice still reinstate in response to other stressors, head restraint does not prohibit investigation of stress effects on reward seeking. Altogether, the influence of restraint-related stress should be acknowledged and considered when designing drug self-administration experiments in head-restrained animals. Moreover, future adaptations of the assay could improve its strength for studying the neural circuit underpinnings of SUD.

## Conclusion

By coupling multiphoton imaging with simultaneous intravenous drug self-administration, we can characterize and manipulate adaptations in neuronal circuits that evolve from the onset of drug use to relapse. The replication of effects observed in freely moving animals indicates that our head-restrained approach could build upon, rather than diverge from, decades of foundational research provided by freely moving drug self-administration studies. Most importantly, this could accelerate discovery of novel therapeutic interventions for SUD.

## Data Availability Statement

The raw data supporting the conclusions of this article will be made available by the authors, without undue reservation.

## Ethics Statement

The animal study was reviewed and approved by Institutional Animal Care and Use Committee (IACUC).

## Author Contributions

KV, ED, and JO designed the experiments and wrote the manuscript. KV, ED, RG, KW, ER, CB, PS, LG, and IT-P performed the experiments. AB and PK provided intellectual support and training for self-administration studies. All authors contributed to the article and approved the submitted version.

## Conflict of Interest

The authors declare that the research was conducted in the absence of any commercial or financial relationships that could be construed as a potential conflict of interest.

## Publisher’s Note

All claims expressed in this article are solely those of the authors and do not necessarily represent those of their affiliated organizations, or those of the publisher, the editors and the reviewers. Any product that may be evaluated in this article, or claim that may be made by its manufacturer, is not guaranteed or endorsed by the publisher.

## Supplementary Material

Supplementary Figure 1 Head-restrained drug self-administration behavioral chamber. *Left:* each two-door cabinet has soundproofing foam **(1)** lining the doors and walls. An infusion pump **(2)** sits atop each behavioral chamber and has tubing extending to the restrained mouse (mouse not shown) **(3)**, allowing i.v. infusions. Each behavioral chamber is equipped with a laptop **(4)**, which interfaces with the Arduino circuit board to control equipment and record behavior. *Right:* Electronics, levers, and the head-fixation station are secured to an aluminum breadboard **(5)** within each behavioral chamber. The head-fixation station **(6)** holds the head-ring which is surgically implanted onto the mouse’s skull. Once the mouse is secured within the head-fixation station, its body is placed within the restraint tube. The mouse can use its forelimbs to press the operant levers **(7)**. When the active lever is pressed, the speaker **(8)** can deliver an auditory cue during acquisition and cue-induced reinstatement sessions. The Arduino circuit board **(9)** and associated electronic breadboards interface with the computer to record behavioral events, as well as control other aspects of the session (e.g., auditory cues, infusions, and session length).

Supplementary Figure 2 Sex comparison across head-fixed drug and sucrose self-administration experiments. *Heroin self-administration.* **(A)** Male and female mice did not differ in the number of i.v. heroin infusions received during acquisition. However, grouped data shows heroin self-administering mice received more infusions as compared with the saline self-administering mice (*p* < 0.001). **(B)** Male and female mice did not differ in active lever pressing rates during extinction of heroin seeking. **(C)** Male and female mice did not display differences during cue-, heroin-, yohimbine-, predator odor (TMT)-, or saline-induced reinstatement tests. *Cocaine self-administration.* **(D)** Male and female mice did not differ in the number of i.v. cocaine infusions received during acquisition. However, grouped data shows cocaine self-administering mice received more infusions as compared with the saline self-administering mice (*p* < 0.001). **(E)** Male and female mice did not differ in active lever pressing rates during extinction of cocaine seeking. **(F)** Male and female mice did not display differences during cue-, cocaine-, yohimbine-, or predator odor (TMT)-induced reinstatement tests. However, males pressed the active lever significantly more than females during the saline control test (*p* = 0.014). *Sucrose self-administration.* **(G)** Male and female mice did not differ in the number of sucrose droplets received during acquisition. **(H)** Male and female mice did not differ in active lever pressing rates during extinction of sucrose seeking. **(I)** Male and female mice did not display differences during cue-, heroin-, yohimbine-, predator odor (TMT)-, or saline-induced reinstatement tests.

Supplementary Figure 3 Inactive lever pressing across head-fixed drug and sucrose self-administration experiments. *Heroin self-administration.* **(A)** Mice showed no difference in inactive lever pressing across extinction. **(B)** Mice showed no change in inactive lever pressing from extinction to cue-, heroin-, yohimbine-, predator odor (TMT)-, or saline-induced reinstatement. *Cocaine self-administration.* **(C)** Mice significantly decreased inactive lever pressing from the first day of extinction to the last. **(D)** Mice showed no change in inactive lever pressing from extinction to cue-, cocaine-, yohimbine-, predator odor (TMT)-, or saline-induced reinstatement. *Sucrose self-administration.* **(E)** Mice showed no difference in inactive lever pressing across extinction. **(F)** Mice showed no change in inactive lever pressing from extinction to cue-, heroin-, yohimbine-, predator odor (TMT)-, or saline-induced reinstatement.

Supplementary Figure 4 Active lever pressing across extinction and reinstatement testing for saline self-administration experiments. **(A)** Due to low lever responding across acquisition, saline self-administering mice did not decrease active lever pressing from the first day of extinction to the last. **(B)** Saline self-administering mice did not increase active lever pressing to cue-, heroin-, yohimbine-, or saline-induced reinstatement tests.
