# Supplementary figures and images for: A Novel Assay Allowing Drug Self-Administration, Extinction, and Reinstatement Testing in Head-Restrained Mice
Source: Front Behav Neurosci. 2021 Oct 29;15:744715. doi: 10.3389/fnbeh.2021.744715 (PMC8585999; doi:10.3389/fnbeh.2021.744715)

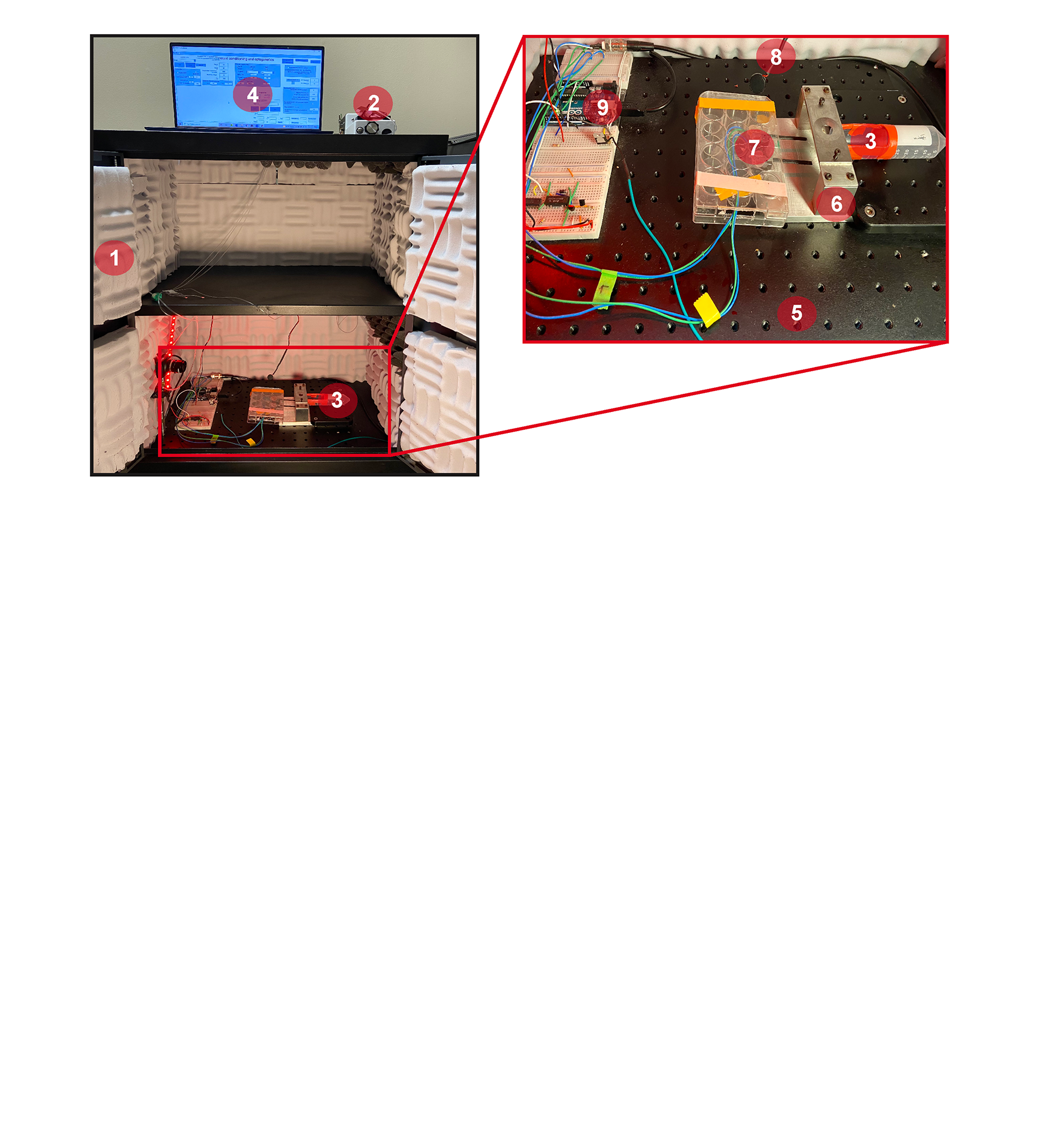

Supplement: Supplementary Figure 1 — Head-restrained drug self-administration behavioral chamber. Left: each two-door cabinet has soundproofing foam (1) lining the doors and walls. An infusion pump (2) sits atop each behavioral chamber and has tubing extending to the restrained mouse (mouse not shown) (3), allowing i.v. infusions. Each behavioral chamber is equipped with a laptop (4), which interfaces with the Arduino circuit board to control equipment and record behavior. Right: Electronics, levers, and the head-fixation station are secured to an aluminum breadboard (5) within each behavioral chamber. The head-fixation station (6) holds the head-ring which is surgically implanted onto the mouse’s skull. Once the mouse is secured within the head-fixation station, its body is placed within the restraint tube. The mouse can use its forelimbs to press the operant levers (7). When the active lever is pressed, the speaker (8) can deliver an auditory cue during acquisition and cue-induced reinstatement sessions. The Arduino circuit board (9) and associated electronic breadboards interface with the computer to record behavioral events, as well as control other aspects of the session (e.g., auditory cues, infusions, and session length). [file Image_1.tiff]

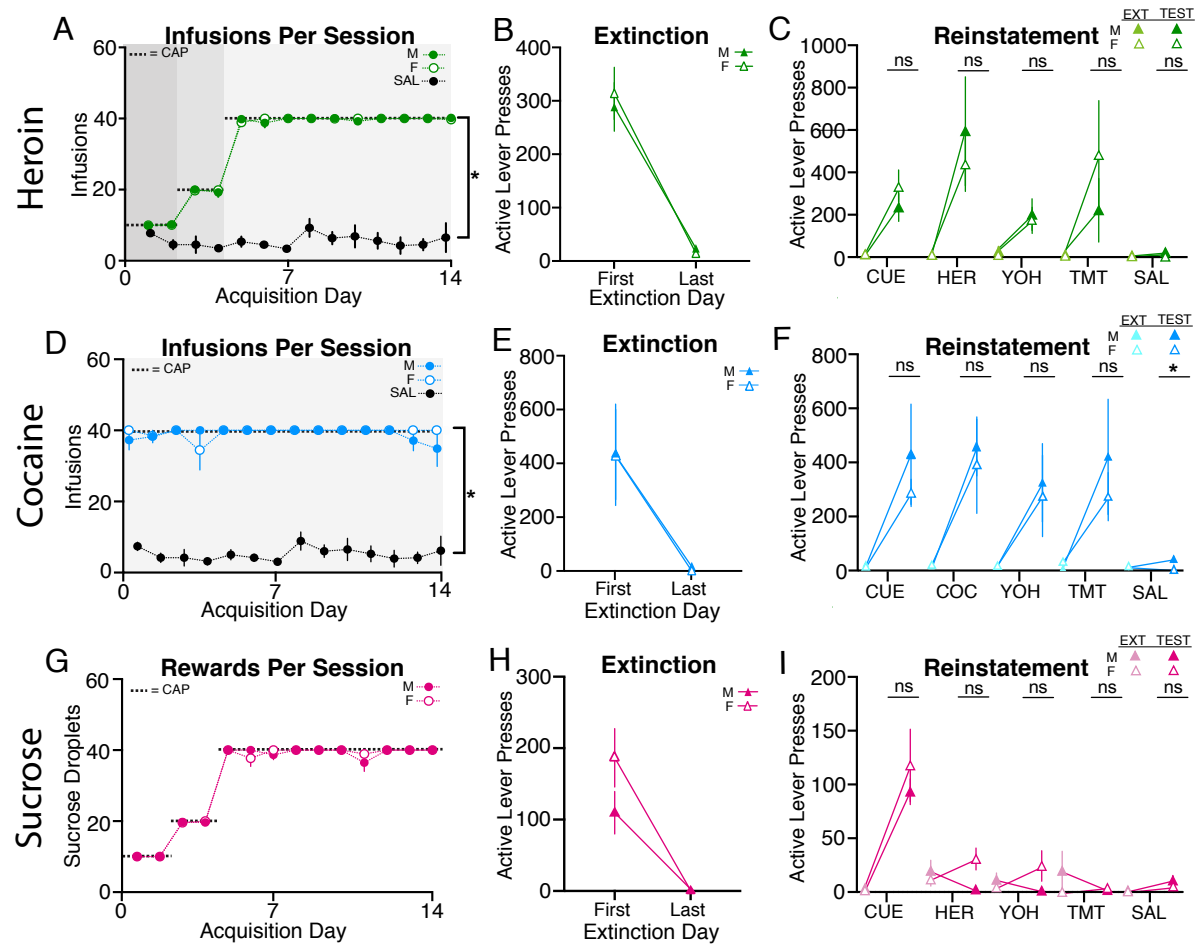

Supplement: Supplementary Figure 2 — Sex comparison across head-fixed drug and sucrose self-administration experiments. Heroin self-administration. (A) Male and female mice did not differ in the number of i.v. heroin infusions received during acquisition. However, grouped data shows heroin self-administering mice received more infusions as compared with the saline self-administering mice (p < 0.001). (B) Male and female mice did not differ in active lever pressing rates during extinction of heroin seeking. (C) Male and female mice did not display differences during cue-, heroin-, yohimbine-, predator odor (TMT)-, or saline-induced reinstatement tests. Cocaine self-administration. (D) Male and female mice did not differ in the number of i.v. cocaine infusions received during acquisition. However, grouped data shows cocaine self-administering mice received more infusions as compared with the saline self-administering mice (p < 0.001). (E) Male and female mice did not differ in active lever pressing rates during extinction of cocaine seeking. (F) Male and female mice did not display differences during cue-, cocaine-, yohimbine-, or predator odor (TMT)-induced reinstatement tests. However, males pressed the active lever significantly more than females during the saline control test (p = 0.014). Sucrose self-administration. (G) Male and female mice did not differ in the number of sucrose droplets received during acquisition. (H) Male and female mice did not differ in active lever pressing rates during extinction of sucrose seeking. (I) Male and female mice did not display differences during cue-, heroin-, yohimbine-, predator odor (TMT)-, or saline-induced reinstatement tests. [file Image_2.pdf]

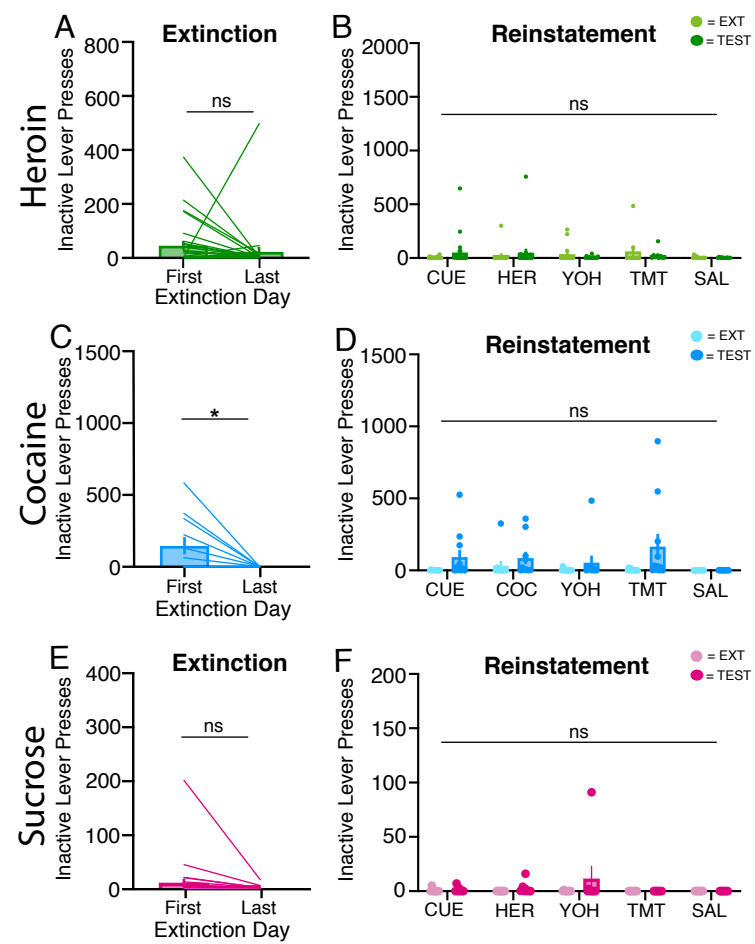

Supplement: Supplementary Figure 3 — Inactive lever pressing across head-fixed drug and sucrose self-administration experiments. Heroin self-administration. (A) Mice showed no difference in inactive lever pressing across extinction. (B) Mice showed no change in inactive lever pressing from extinction to cue-, heroin-, yohimbine-, predator odor (TMT)-, or saline-induced reinstatement. Cocaine self-administration. (C) Mice significantly decreased inactive lever pressing from the first day of extinction to the last. (D) Mice showed no change in inactive lever pressing from extinction to cue-, cocaine-, yohimbine-, predator odor (TMT)-, or saline-induced reinstatement. Sucrose self-administration. (E) Mice showed no difference in inactive lever pressing across extinction. (F) Mice showed no change in inactive lever pressing from extinction to cue-, heroin-, yohimbine-, predator odor (TMT)-, or saline-induced reinstatement. [file Image_3.pdf]

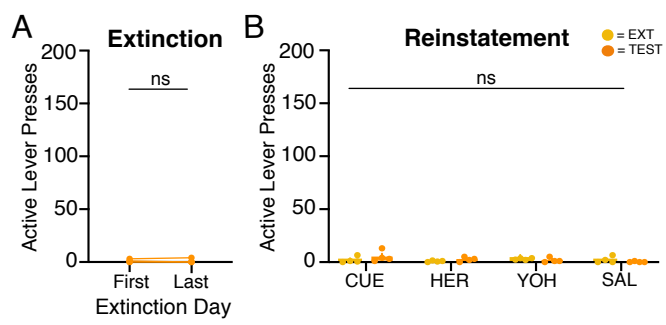

Supplement: Supplementary Figure 4 — Active lever pressing across extinction and reinstatement testing for saline self-administration experiments. (A) Due to low lever responding across acquisition, saline self-administering mice did not decrease active lever pressing from the first day of extinction to the last. (B) Saline self-administering mice did not increase active lever pressing to cue-, heroin-, yohimbine-, or saline-induced reinstatement tests. [file Image_4.pdf]
